# Closing the Gap for Electronic Short‐Circuiting: Photosystem I Mixed Monolayers Enable Improved Anisotropic Electron Flow in Biophotovoltaic Devices

**DOI:** 10.1002/anie.202008958

**Published:** 2020-11-23

**Authors:** Panpan Wang, Anna Frank, Fangyuan Zhao, Julian Szczesny, João R. C. Junqueira, Sónia Zacarias, Adrian Ruff, Marc M. Nowaczyk, Inês A. C. Pereira, Matthias Rögner, Felipe Conzuelo, Wolfgang Schuhmann

**Affiliations:** ^1^ Analytical Chemistry—Center for Electrochemical Sciences (CES) Faculty of Chemistry and Biochemistry Ruhr University Bochum Universitätsstrasse 150 44780 Bochum Germany; ^2^ Plant Biochemistry Faculty of Biology and Biotechnology Ruhr University Bochum Universitätsstrasse 150 44780 Bochum Germany; ^3^ Instituto de Tecnologia Química e Biológica António Xavier Universidade Nova de Lisboa Oeiras 2780-157 Portugal; ^4^ Present Address: PPG (Deutschland) Business Support GmbH PPG Packaging Coatings EMEA Erlenbrunnenstrasse 20 72411 Bodelshausen Germany

**Keywords:** Biophotovoltaics, Electrochemistry, Langmuir–Blodgett films, Photosystem I, Redox polymers

## Abstract

Well‐defined assemblies of photosynthetic protein complexes are required for an optimal performance of semi‐artificial energy conversion devices, capable of providing unidirectional electron flow when light‐harvesting proteins are interfaced with electrode surfaces. We present mixed photosystem I (PSI) monolayers constituted of native cyanobacterial PSI trimers in combination with isolated PSI monomers from the same organism. The resulting compact arrangement ensures a high density of photoactive protein complexes per unit area, providing the basis to effectively minimize short‐circuiting processes that typically limit the performance of PSI‐based bioelectrodes. The PSI film is further interfaced with redox polymers for optimal electron transfer, enabling highly efficient light‐induced photocurrent generation. Coupling of the photocathode with a [NiFeSe]‐hydrogenase confirms the possibility to realize light‐induced H_2_ evolution.

## Graphical abstract

Towards the development of improved biophotovoltaic devices for solar energy conversion, a mixed monolayer constituted by photosystem I trimers and monomers enables the fabrication of highly efficient biophotoelectrodes by minimizing electronic short‐circuiting processes while at the same time ensuring a high density of photoactive molecules per unit area.

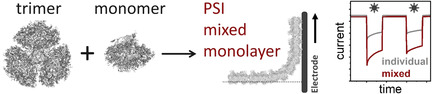

## Introduction

**Figure 1:**
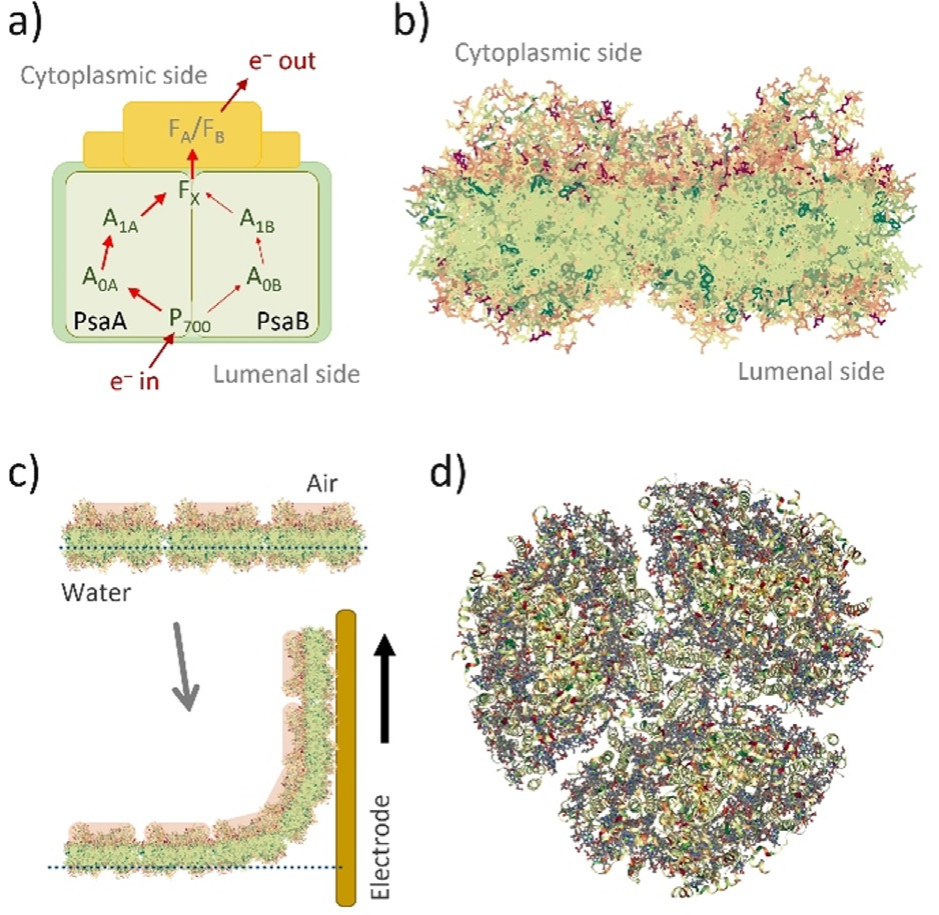
a) Schematic representation of the electron transport pathway in PSI. The cofactors include chlorophyll molecules (P_700_, A_0_), phylloquinones (A_1_), and Fe–S clusters (F_X_, F_A_, F_B_). PsaA and PsaB: protein subunits.^[14]^ b) Side view of the protein complex highlighting its hydrophobic core (green) and the two hydrophilic ends (red) where the terminal redox sites are located. c) PSI monolayer at the air/water interface and Langmuir–Blodgett film transfer onto the electrode surface. d) Top view of trimeric cyanobacterial PSI. PDB ID: 4FE1.^[15]^ Protein images created using NLG viewer (https://doi.org/10.1093/bioinformatics/bty419), RCSB PDB (rcsb.org).

In the development of solar energy conversion devices, the use of photosynthetic protein complexes stands as an interesting possibility due to the high quantum efficiency and the abundant occurrence of these biomolecules in nature. In consequence, several strategies have shown the possible implementation of semi‐artificial devices by integration of isolated protein complexes with electrodes.^[1]^ In particular, photosystem I (PSI) is a robust biomolecule able to perform charge separation upon visible light absorption and has inspired the fabrication of different biophotovoltaic devices.[^2^, ^3^] After sequential internal electron transfer in PSI (Figure 1 a), an intermediate state with a relatively long lifetime in the millisecond timescale^[4]^ is obtained, consisting of two terminal redox centers of opposite charge: the photo‐oxidized special chlorophyll pair (P_700_^+^) and the reduced terminal Fe–S cluster (F_B_^−^), with a voltage difference of approx. 1 V.^[5]^ The high‐energy electrons exiting PSI at a potential of −580 mV vs. SHE can be eventually used in reductive processes of interest, including the production of fuels such as H_2_.[^6^, ^7^] Nevertheless, integration of PSI with electrodes is extremely challenging. The implementation of PSI‐based biodevices for energy conversion purposes has been mainly hampered by a short operational stability and a relatively low efficiency in the extraction of high‐energy electrons.^[8]^ In order to tackle these constraints, different studies have shown that a rational operation of PSI‐based bioelectrodes can lead to significantly improved stability.^[9]^ However, the biggest inherent limitation is associated with the high voltage difference between the terminal redox centers at PSI, imposing a large driving force for recombination processes and short‐circuiting due to re‐oxidation of reduced charge carriers.^[10]^ As a result, the generated photocurrents are partially cancelled out compromising energy conversion efficiency. Therefore, the implementation of advanced structures capable of providing unidirectional electron flow and enabling the large potential separation generated at PSI to be exploited is highly important.

In nature, PSI is embedded in the thylakoid membrane that acts as a barrier for preventing possible short‐circuiting pathways between electron donors and acceptors. In analogy, the fabrication of bioelectrodes by reconstitution of PSI into lipid bilayers has demonstrated formation of highly packed structures preventing short‐circuiting processes and leading to photocurrents of up to one order of magnitude larger in comparison to dense PSI monolayers.^[11]^ However, this strategy limits the number of photoactive elements per unit area. Moreover, since the terminal redox centers are located at opposite sides of the protein complex, controlled orientation of immobilized PSI is also a relevant factor for an ultimately effective extraction of high‐energy electrons (Figure 1 a). Taking advantage of the amphiphilic nature of PSI, we have recently shown the possibility for fabrication of monolayer structures revealing a preferential orientation of the immobilized PSI protein complexes using Langmuir–Blodgett films.^[12]^ PSI protein complexes exhibit a hydrophobic core and two hydrophilic ends where the terminal redox sites are located (Figure 1 b). As it has been shown before, isolated PSI protein complexes adopt a specific orientation at the air/water interface,^[13]^ with the lumenal side preferentially facing water and the stromal or cytoplasmic side mainly facing air. This enables the formation of a monolayer structure by stacking of the hydrophobic regions. The film can then be transferred to an electrode surface retaining the compact structure and defined orientation as at the air/water interface, ensuring accessibility of the redox centers involved in light‐driven electron transfer processes (Figure 1 c). The use of trimeric PSI monolayers further interfaced with adequately designed electron donors and acceptors allowed the fabrication of biophotoelectrodes with diminished short‐circuiting pathways and hence increased photocurrents, making even the implementation of a fully light‐driven Z‐scheme mimic biophotovoltaic cell for bias‐free water splitting possible.^[12]^

Cyanobacterial PSI naturally occurs as a mixture of monomers and trimers in the thylakoid membrane.[^16^, ^17^] The prevailing structure is a trimeric disk‐shaped oligomer (Figure 1 d) with dimensions of approx. 9 nm in height and 22 nm in diameter.^[18]^ Evidently, a close packing of PSI trimers can be associated with the inherent existence of gaps within a monolayer structure. Accordingly, the estimated surface coverage with PSI trimers constituting the Langmuir–Blodgett film deposited on the electrode surface was about 61 %.^[12]^ Therefore, although substantially minimized, residual short‐circuiting processes may occur, limiting the ultimate performance of the bioelectrode. Aiming for a further improved and optimized structure, a mixed monolayer comprised of trimeric and monomeric PSI was envisaged. As it has been suggested by calculations, a combination of PSI trimeric and monomeric structures might lead to an increased surface coverage.^[3]^ This strategy is expected to result in a more compact film preventing re‐oxidation of charge carriers. Importantly, the only molecules involved in the formation of the monolayer are photoactive units, i.e., PSI trimers or monomers. This ensures an increased number of photoactive molecules per unit area and leads to an overall enhanced electron flow.

## Results and Discussion

In order to ensure maximum compatibility of elements constituting the mixed monolayer, the use of PSI monomers and trimers from the same organism was envisaged. Thus, an optimized procedure was used for isolating monomeric PSI from *T. elongatus* (for details see the Supporting Information and Figure S1). To confirm that the isolated PSI monomers remain active, the responses of electrodes cast with either PSI monomers or trimers embedded in a tridimensional matrix of an Os‐complex‐modified redox polymer (P‐Os) were compared. In agreement with previous reports,^[19]^ electrodes prepared with equal chlorophyll concentrations provided similar photocurrents (Figure S2). Furthermore, the feasibility of isolated PSI monomers to form dense Langmuir–Blodgett films was evaluated by constructing a monolayer constituted entirely of isolated monomeric PSI units. However, in this case, the PSI monomer‐monolayer modified electrodes did not provide any detectable photocurrents. A possible reason for the obtained result was found in the use of lauryldimethylamine oxide (LDAO, Figure S3a) as surfactant for passivation of the exposed hydrophobic surfaces during PSI monomer isolation. The ionic detergent hampered the re‐association of monomers but also provided an increased hydrophilicity to the isolated protein complexes hence increasing their solubility and with this preventing suspension of PSI at the air/water interface and Langmuir monolayer formation. After exchange of the surfactant for *n*‐dodecyl‐β‐d‐maltoside (DDM, Figure S3b; for details, see the Supporting Information), monolayers constituted entirely of PSI monomers could be successfully formed and transferred to the electrode surface, leading to the effective generation of photocurrents (Figure S4), as observed before for monolayers constituted entirely by PSI in trimeric form.

**Figure 2:**
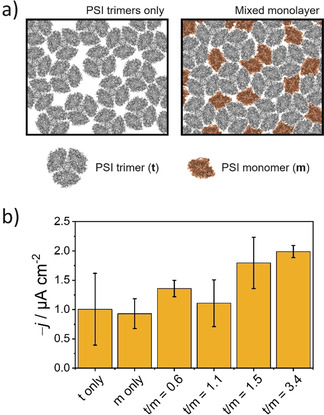
a) Schematic representation showing the proposed improvement in surface coverage with PSI monolayers comprised of a mixture of trimers and monomers in comparison with a monolayer constituted by trimeric complexes only. b) Mean photocurrent recorded for monolayers prepared using either PSI trimers (t), PSI monomers (m) or mixtures as indicated (ratios of μg_Chl_ used of each PSI preparation). PSI monolayers deposited on Au substrates. Transfer pressure for PSI_t_ 42 mN m^−1^, for PSI_m_ 29 mN m^−1^, for mixed monolayers 32 mN m^−1^. *E*_app_=210 mV vs. SHE. Illumination with red light at an incident power of 51 mW cm^−2^. Electrolyte: 2 mm MV^2+^ in air‐equilibrated 150 mm phosphate‐citrate buffer, pH 4.0. Error bars represent the standard deviation (*N*=3).

For the intended fabrication of mixed compositions providing an improved surface coverage with photoactive elements (Figure 2 a) and in order to find an optimal ratio between PSI monomers and trimers, various monolayers were prepared using different ratios of the two PSI forms. The differently prepared mixed monolayers were then transferred onto Au substrates and compared with monolayers constituted entirely of PSI monomers or PSI trimers. The preferential orientation of isolated PSI at the air/water interface leads to the P_700_ redox center (located at the lumenal side) directing towards the electrode surface upon pulling the electrode substrate during monolayer transfer (Figure 1 c), making the fabrication of a photocathode possible. The photocurrent response was measured for each case with the immobilized PSI monolayer in direct electron transfer with the electrode surface and in the presence of methyl viologen (MV^2+^) as free‐diffusing electron scavenger in solution and O_2_ as terminal electron acceptor.^[20]^

An initial comparison of the responses obtained for monolayers constituted only by either monomeric PSI or trimeric PSI revealed photocurrents of comparable magnitude (Figure 2 b). A priori, a denser packing is possible for a monolayer incorporating only PSI monomer units and a larger response would be expected. However, the obtained results could be explained by the previously described cooperative energy transfer achieved between adjacent PSI monomers associated within a trimer complex^[19]^ leading to a higher photochemical activity of PSI trimers, particularly under red light illumination.^[16]^ In addition, in contrast to PSI monomers, the larger dipole moment of PSI trimers^[19]^ might be responsible for an improved anisotropic orientation at the air/water interface, translating into a more ordered monolayer structure.

The use of mixed monolayers delivered higher photocurrent densities in comparison with those obtained for monolayers constituted by only trimeric or monomeric PSI. Furthermore, a more reproducible film formation was observed. In order to confirm the formation of a more compact structure for mixed monolayers, electrochemical impedance spectroscopy measurements were performed in the presence of [Fe(CN)_6_]^4−^/[Fe(CN)_6_]^3−^ as redox probe (Figure S5). A considerably higher charge transfer resistance was observed in case of the mixed use of PSI monomers and trimers. Hence, confirming the formation of a more densely packed monolayer structure leading to minimal short‐circuiting processes and, at the same time, a higher density of photoactive molecules per unit area, which in turn contributed to an overall enhanced photocurrent response. The optimal mixed monolayer composition with the highest performance of photocurrents of about −2.0 μA cm^−2^ was selected for further experiments. The use of mixed monolayers enabled a better control of the film during transfer onto the electrode surface, providing a reproducible and homogeneous fabrication of even relatively large surface area electrodes (>2 cm^2^). This is reflected as well in an improved reproducibility in the photocurrent response (Figure 2 b). As an estimation about the number of immobilized PSI molecules, the modified electrode surfaces were extracted with methanol for determining the chlorophyll loading (see Supporting Information for details). The obtained loading for electrodes modified with a mixed PSI monolayer was (42±17) ng_Chl_ cm^−2^ (*N*=3).

**Figure 3:**
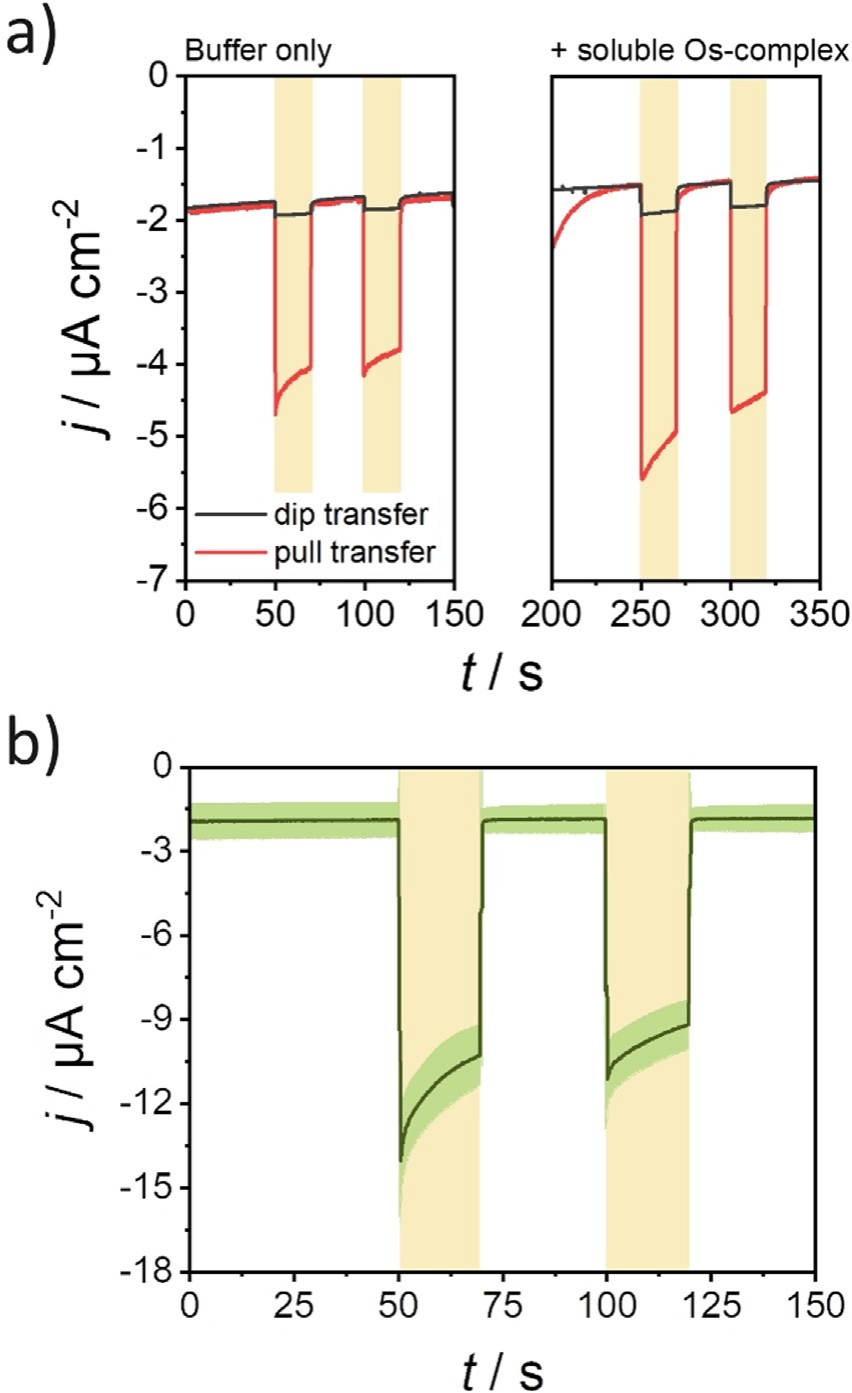
a) Photocurrent response for mixed monolayers on electrodes (the direction of monolayer transfer is indicated) before (left) and after (right) the addition of 125 μm soluble Os complex. b) Average photocurrent response for mixed monolayer deposited on Au substrates first modified with P‐Os. The green shaded region indicates the standard deviation of the measurements (*N*=3). For both panels: Electrolyte: 2 mm MV^2+^ in air‐equilibrated 150 mm phosphate‐citrate buffer, pH 4.0. *E*_app_=210 mV vs. SHE. Illumination with red light (51 mW cm^−2^) during the times indicated by the yellow boxes.

To confirm the anisotropic orientation of PSI monomers and trimers constituting the mixed monolayer, the photocurrent responses generated by electrodes incorporating PSI mixed monolayers prepared by either pulling or dipping the electrode substrate during monolayer transfer were compared (Figure 3 a, left). Substantial cathodic photocurrents were obtained for monolayers deposited by pulling the electrode during transfer. As this situation leads to the PSI side facing water (lumenal side) to be located adjacent to the electrode surface (Figure 1 c), this orientation enables the reduction of the photo‐oxidized P_700_^+^ site by injection of electrons from the electrode surface. On the contrary, electrodes fabricated by dipping the electrode substrate during monolayer transfer caused immobilization of most PSI with the cytoplasmic side located in close proximity to the electrode surface. Thus, electron transfer to the lumenal side at PSI is mostly prevented, as reflected by only a small residual cathodic photocurrent. The obtained results confirmed a preferential orientation of PSI protein complexes within the film formed at the air/water interface, allowing the fabrication of bioelectrodes with an anisotropic electron flow.

The photocurrent response for both kinds of electrodes was also evaluated after the addition of [Os‐(1‐(*n*‐butyl)‐imidazole)‐(2,2′‐bipyridine)_2_Cl]Cl, a freely diffusing Os‐complex with a redox potential of about 400 mV vs. SHE,^[20]^ able to act as a redox mediator between the electrode surface and the P_700_^+^ site at PSI. In the case of a PSI trimer monolayer incorporation of the soluble Os‐complex into the electrochemical cell was associated to an increased photocurrent. This was observed independently of the direction of transfer during monolayer deposition and was caused by the fact that the obtained monolayer structure does not completely block the electrode surface underneath.^[12]^ In comparison, the results obtained with a mixed monolayer (Figure 3 a, right) confirmed the formation of highly compact films as only a minor increase in photocurrent was observed after addition of the soluble redox mediator. In the presence of a highly compact monolayer, the diffusion of Os‐complexes towards the underlying electrode surface was prevented. Therefore, for the mixed monolayer consisting of PSI with the lumenal side directing to the electrode surface, only a slight increase in photocurrent was observed upon addition of the Os‐complex. The average increase in photocurrent response observed for three similarly prepared different electrodes was (1.8±0.9) times. In comparison, for a PSI trimer monolayer the increase in photocurrent was more than 6 times.^[12]^ Moreover, for electrodes fabricated with the PSI cytoplasmic side directed to the electrode surface, a photocurrent increase of about two times was observed after addition of the Os‐complex, in comparison with more than 10 times higher photocurrents observed for a PSI trimer monolayer.^[12]^

The optimized mixed monolayer was further interfaced in a layered assembly with redox polymers able to provide an improved electrical communication with PSI. First, for a more efficient wiring between the electrode surface and the photosynthetic protein complexes, the redox polymer poly(1‐vinylimidazole‐*co*‐allylamine)‐[Os(2,2′‐bipyridine)_2_Cl]Cl (P‐Os) was used, a redox mediator suitable for the transfer of electrons to the P_700_^+^ site.[^20^, ^21^] Therefore, prior to monolayer transfer, the electrode surface was modified with a P‐Os film, with an estimated surface coverage of electrochemically active redox centers of (62±28) pmol cm^−2^ (*N*=3). Importantly, the P‐Os film was also confirmed to be sufficiently homogeneous (see Figure S6), making the deposition of the PSI monolayer retaining a compact structure and the specific orientation of constituting PSI units possible. The performance of the assembly was evaluated in the presence of MV^2+^ as electron acceptor in solution. In this case, the observed photocurrent was −(9±1) μA cm^−2^ (*N*=3, Figure 3 b), which is about 4.5 times higher than the response obtained under direct electron transfer conditions (−(2.0±0.2) μA cm^−2^, Figure 2 b). These values are among the highest photocurrents reported for a PSI monolayer on semiconductor‐free electrodes.^[12]^

**Figure 4:**
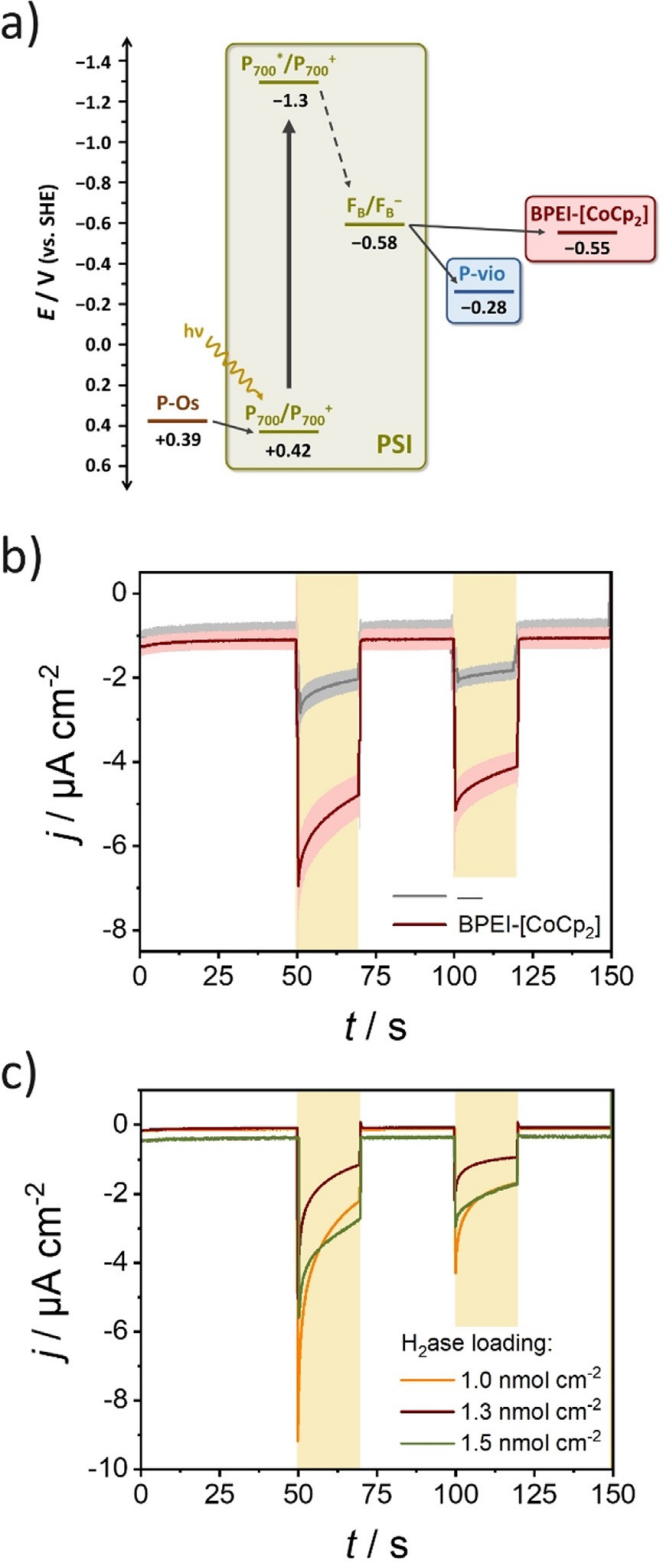
a) Energy level diagram for the cofactors involved in light‐induced charge separation and electron transfer at PSI and midpoint potentials of the redox polymers used in this study. b) Average photocurrent response for PSI‐LB/P‐Os/Au in the absence or presence of a top modification layer consisting of BPEI‐[CoCp_2_] (loading: 165 μg cm^−2^). The shaded regions indicate the standard deviation of the measurements (*N*=3). Electrolyte: air‐equilibrated 150 mm phosphate‐citrate buffer, pH 5.6. *E*_app_=210 mV vs. SHE. Illumination with red light (51 mW cm^−2^) during the times indicated by the yellow boxes. c) Photocurrent response for PSI‐LB/P‐Os/Au with a top H_2_ase/BPEI‐[CoCp_2_] layer, nominal loadings of embedded H_2_ase indicated in the Figure, redox polymer loading: 165 μg cm^−2^. Electrolyte: Ar‐saturated 150 mm phosphate‐citrate buffer, pH 5.6. *E*_app_=210 mV vs. SHE. Illumination with white light (113 mW cm^−2^) during the times indicated by the yellow boxes.

Subsequently, in order to replace the use of a freely diffusing electron scavenger, such as MV^2+^, for the uptake of high‐energy electrons exiting PSI, a polymer‐bound low‐potential redox mediator was implemented as a top modification layer. As it was shown before, this strategy further decreases the possibility for short‐circuiting processes by confinement of the electron scavenger in a polymer layer.^[12]^ Two different redox polymers were evaluated, namely a viologen‐modified polymer (poly(3‐azidopropyl methacrylate‐*co*‐butyl acrylate‐*co*‐glycidyl methacrylate)‐viologen, P‐vio) and a cobaltocene‐modified polymer (cobaltocene‐functionalized branched polyethyleneimine, BPEI‐[CoCp_2_], Scheme S1). The selected redox polymers were chosen as they are capable of providing electrical wiring with enzymes performing reductive reactions of interest.^[22]^ This has been shown recently for P‐vio in combination with hydrogenases,[^12^, ^23^] as well as for different cobaltocene‐modified polymers in combination with hydrogenases,[^7^, ^24^] formate dehydrogenase,^[25]^ diaphorase for NADH regeneration purposes,^[26]^ and nitrogenase.^[27]^ The photocurrent response was measured using ambient O_2_ as terminal electron acceptor, with the polymer‐bound redox centers enabling an efficient electron transfer with the terminal Fe–S cluster at PSI. Moreover, considering the possibility for coupling of the assembly with a hydrogenase for realizing light‐induced H_2_ evolution, the characterization was performed at a pH value of 5.6, optimal for the simultaneous operation of both biomolecules.^[28]^ In contrast to P‐vio, with a midpoint potential of −280 mV vs. SHE,^[12]^ BPEI‐[CoCp_2_] presents a more negative midpoint potential of about −550 mV vs. SHE (Figure S7), closer to the formal potential of the terminal F_B_ site at PSI (Figure 4 a). The possibility for effective electron transfer between the PSI mixed monolayer and the low‐potential redox polymers was evaluated by comparing the photocurrent response for PSI monolayer/P‐Os assemblies in the absence and presence of a top modification layer constituted of P‐vio or BPEI‐[CoCp_2_]. An increased photocurrent response was observed when P‐vio was incorporated as a top modification layer (Figure S8a), in agreement with similar results obtained before for a PSI monolayer constituted entirely of trimeric units.^[12]^ The smaller potential difference between the terminal redox site at PSI and BPEI‐[CoCp_2_] in comparison with P‐vio (Figure 4 a) translates into a lower driving force for the transfer of electrons exiting PSI. Therefore, it was important to investigate the possibility for a productive electron transfer using the cobaltocene‐modified polymer. Significantly larger photocurrents in the presence of the top modification layer (Figure 4 b) confirmed that BPEI‐[CoCp_2_] can also be used as a suitable electron acceptor for the high‐energy electrons exiting the terminal Fe–S cluster at PSI.

The low‐potential redox polymer top modification layer constituted a suitable matrix for the immobilization and electrical wiring of (bio)catalysts able to perform reductive reactions of interest. Thus, as a proof of concept, the assembly was interfaced with a [NiFeSe]‐hydrogenase from *Desulfovibrio vulgaris* Hildenborough (H_2_ase).[^23^, ^29^] The effective electronic communication between H_2_ase and the redox polymer matrix enabling H_2_ evolution has been shown before for P‐vio^[12]^ and was also confirmed for BPEI‐[CoCp_2_] (Figure S9). After incorporation of the H_2_ase into the PSI‐based bioelectrode, the occurrence of cathodic photocurrents under Ar‐saturated conditions was observed, either using P‐vio (see Figure S8b) or BPEI‐[CoCp_2_] (Figure 4 c). The evaluation of bioelectrodes with an increased H_2_ase loading in the latter case did not translate directly into a noticeably increased photocurrent response. Due to the complex multi‐parameter system, with interplays between electron transfer within the redox polymer, dilution of the redox polymer by the non‐conducting enzyme, light absorption of the polymer film itself and local pH values during the reaction, a simple optimization is not straightforward. Nevertheless, the possibility for light‐induced H_2_ evolution with the proposed bioelectrodes was confirmed by analyzing the headspace of a small‐volume electrochemical cell by means of gas chromatography (for details, see the Supporting Information). The obtained results after an irradiation time of about 36 min (Figure S10) confirmed the occurrence of light‐induced H_2_ evolution.

## Conclusion

Isolated cyanobacterial PSI in monomeric and trimeric forms have been used for the fabrication of mixed Langmuir–Blodgett films over the electrode surface. The use of mixed PSI monolayers makes the formation of more compact and ordered structures possible, in contrast to monolayers constituted entirely by trimeric PSI. This translates into an increased photocurrent response from about −1.0 μA cm^−2^ for a monolayer constituted entirely by PSI trimers to −(2.0±0.2) μA cm^−2^ for the mixed monolayer with an optimal composition. The increased performance and reproducibility of PSI mixed monolayer‐based bioelectrodes is attributed to decreased short‐circuiting processes as a result of a more densely packed structure. Moreover, in contrast to previous reports where additional non‐photoactive components are used for the formation of highly packed structures, the only elements involved in monolayer formation here are functional PSI complexes, ensuring a high density of photoactive molecules per unit area. Additional incorporation of an Os‐complex‐modified redox polymer for a more efficient electrical wiring between the electrode surface and the immobilized PSI film translates into photocurrent responses of up to −(9±1) μA cm^−2^. Furthermore, the assembly was effectively coupled to low‐potential redox polymers for the uptake of high‐energy electrons exiting PSI. In addition to the use of a viologen‐modified polymer, the possibility of coupling of the proposed PSI‐based bioelectrode with a cobaltocene‐modified polymer has also been confirmed. As this latter redox polymer exhibits a more negative midpoint potential in comparison to the viologen‐modified polymer shown before, this strategy could open the possibility for coupling of PSI to other (bio)catalysts of interest requiring high‐energy electrons (e.g., formate dehydrogenase and nitrogenase) that can thus be generated under irradiation of the biophotoelectrode. As a proof of concept, the system has been coupled to a hydrogenase for realizing light‐induced hydrogen evolution. Future work will be directed to an even more efficient coupling between the PSI monolayer and integrated biocatalysts, towards the implementation of practical solar energy conversion biodevices.

## Conflict of interest

The authors declare no conflict of interest.

## Supporting information

As a service to our authors and readers, this journal provides supporting information supplied by the authors. Such materials are peer reviewed and may be re‐organized for online delivery, but are not copy‐edited or typeset. Technical support issues arising from supporting information (other than missing files) should be addressed to the authors.

Supplementary
