# Supplementary material for: Closing the Gap for Electronic Short‐Circuiting: Photosystem I Mixed Monolayers Enable Improved Anisotropic Electron Flow in Biophotovoltaic Devices
Source: Angew Chem Int Ed Engl. 2020 Nov 23;60(4):2000–6. doi: 10.1002/anie.202008958 (PMC7894356; doi:10.1002/anie.202008958)
Supplement: Supplementary file 1 — Supplementary [file ANIE-60-2000-s001.pdf]

Angewandte Chemie
Eine Zeitschrift der Gesellschaft Deutscher Chemiker

Supporting Information

Closing the Gap for Electronic Short-Circuiting: Photosystem I Mixed Monolayers Enable Improved Anisotropic Electron Flow in Biophotovoltaic Devices

Panpan Wang, Anna Frank, Fangyuan Zhao, Julian Szczesny, João R. C. Junqueira, Sónia Zacarias, Adrian Ruff, Marc M. Nowaczyk, Inês A. C. Pereira, Matthias Rögner, Felipe Conzuelo, and Wolfgang Schuhmann**

anie_202008958_sm_miscellaneous_information.pdf

# – Supporting Information –

**CONTENTS**	**PAGE**
Experimental Section	S2
PSI monomer preparation	S3
Synthesis of BPEI-[$CoCp_2$]	S4
Additional Figures	S5
References	S8

Experimental Section

Chemicals and Materials

Potassium dihydrogen phosphate, di-potassium hydrogen phosphate trihydrate, and potassium chloride (VWR Chemicals); citric acid monohydrate (J.T. Baker); Tris-HCl (AppliChem); methyl viologen dichloride hydrate (MV^{2+}), branched polyethyleneimine (PEI, average M_n = 600 g mol^{-1}), lauryldimethylamine oxide (LDAO), *n*-dodecyl β-D-maltoside (DDM), potassium ferricyanide, and potassium ferrocyanide (Sigma-Aldrich); poly(ethylene glycol)-diglycidyl ether (PEGDGE, M_n = 400 g mol^{-1}, Polysciences). All chemicals were of reagent grade or higher and used as received. All solutions were prepared using deionized purified water (ρ = 18 MΩ cm).

The soluble Os-complex [Os-(1-(*n*-butyl)-imidazole)-$(bpy)_2Cl$]Cl (bpy = 2,2'-bipyridine) has been synthesized as described before.[1,2] The synthesis and purification of the redox polymer P-Os (poly(1-vinylimidazole-*co*-allyl-amine)-[Os$(bpy)_2Cl$]Cl) is described elsewhere.[3] A detailed description about the synthesis and purification of the viologen-modified polymer poly(3-azidopropyl methacrylate-*co*-butyl acrylate-*co*-glycidyl methacrylate)-viologen (P-vio) can be found in ref.[2] The synthesis of the cobaltocene modified polymer, consisting of a cobaltocene-functionalized branched polyethyleneimine (BPEI-[$CoCp_2$]), has been adapted from ref.[4]

Photosystem I trimers from *Thermosynechococcus elongatus* BP-1 wild type *(T. elongatus*) have been isolated and purified according to refs.[2,5] with a final concentration of 1.85 mg_{Chl} mL^{-1}. Photosystem I monomers also from *T. elongatus* were obtained with a concentration of 6.06 mg_{Chl} mL^{-1}. A detailed description about PSI monomer preparation and purification is given in the following section. The [NiFeSe]-hydrogenase (H_2ase) enzyme from *Desulfovibrio vulgaris* Hildenborough was isolated and purified as described in ref.[6]

The Au electrode substrates were prepared using Si(100) wafers (Wacker) coated sequentially with titanium (as an adhesion layer) and gold by vapor deposition in a metal vaporization setup. Before use, the substrates were cleaned using Piranha solution (concentrated H_2SO_4 mixed with 30 wt.% H_2O_2 in a 3 to 1 volumetric ratio). ***Caution!*** *Piranha solution is a powerful oxidizing agent and highly corrosive. Handle with extreme care.*

Electrode Modification and Characterization

For deposition of thin P-Os films prior to monolayer transfer, the Au substrates were incubated overnight in a solution consisting of a mixture of P-Os (0.122 µg µL^{-1}) and PEGDGE (0.5 µg mL^{-1}) as cross-linker. The surface was then incubated for 30 min in a 50 mM Tris-HCl solution to induce polymer collapse.[1] Finally, the modified substrate was rinsed with water to remove any loosely adsorbed redox polymer.

The surface coverage of electrochemically active redox centers (Γ_{Os}) was estimated from cyclic voltammograms at low scan rate (10 mV s^{-1}, lower scan rates did not yield any noticeable redox peaks), according to the following equation: $\Gamma_{Os} = Q(nFA)^{-1}$, where Q is the charge for the redox conversion of the polymer-bound Os-complexes, n is the number of electrons transferred, F is the Faraday constant, and A is the geometric electrode area.

A Langmuir-Blodgett (LB) trough (5.5 cm × 54.0 cm, KSV Instruments) was used for preparation and transfer of PSI monolayers onto the electrode substrates, as it has been described in detail before.[2] Briefly, a software enabled to control two symmetric barriers for monolayer formation at constant compressing speed and subsequent monolayer transfer at constant surface pressure using a Pt Wilhelmy plate to determine the surface pressure. 5 mM phosphate buffer pH 7.0 was used as subphase, on top of which diluted solutions of PSI trimers, monomers or mixtures were deposited. The PSI solution was spread gently at the air/water interface of the subphase to avoid falling of material into the bulk of the solution. LB transfer was performed at a rate of 5 mm min^{-1} at 20 °C. For more experimental details and a description of the setup used, the reader is referred to ref.[2]

The amount of PSI over the electrode surface was estimated by chlorophyll detection via methanol extraction. For this, three different electrodes modified with PSI LB films (modified surface area: 3.6 cm^2) were incubated with 5 mL methanol in a shaker for 60 min. After subsequent evaporation of the solvent, the extracted chlorophyll for each electrode was resuspended in 30 µL methanol. After sedimentation at 15,000 g, an aliquot of 10 µL was loaded on standard treated MST capillaries (NanoTemper Technologies, Inc.) and chlorophyll fluorescence was measured via Monolith NT.155 (NanoTemper Technologies, Inc.), together with an internal chlorophyll standard, at an excitation power of 1% between 605 nm and 640 nm. The initial fluorescence was detected via a Nano RED detector in a range between 670 nm and 730 nm and used for chlorophyll loading calculations.

Additional modification of the PSI modified substrates with P-vio or BPEI-[$CoCp_2$] was performed by drop casting 25 µL of a solution of the corresponding polymer (P-vio: 1.2 mg mL^{-1}, BPEI-[$CoCp_2$]: 1.62 mg mL^{-1}) and cross-linker agent (for P-vio, PEI: 0.032 vol.%; for BPEI-[$CoCp_2$], PEGDGE: 32 µg mL^{-1}). For modification with H_2ase embedded in P-vio, 25 µL of a mixture of P-vio (1.2 mg mL^{-1}), H_2ase (10 µM), and PEI (0.032 vol.%) were deposited over the PSI modified substrate, corresponding with a H_2ase nominal loading of 1.0 nmol cm^{-2}. For modification with H_2ase embedded in BPEI-[$CoCp_2$], 25 µL of a mixture of P-vio (1.2 mg mL^{-1}), H_2ase (10 µM, 12.5 µM, or 15 µM), and PEGDGE (32 µg mL^{-1}) were deposited over the PSI modified substrate, corresponding with H_2ase nominal

loadings of 1.0 nmol cm^{-2}, 1.3 nmol cm^{-2}, and 1.5 nmol cm^{-2}, respectively. After modification, the substrates were incubated until dry at 4 °C in the dark.

Electrochemical Measurements

A conventional three-electrode setup was used, consisting of the PSI modified substrate as working electrode (with a surface area of 0.246 cm^2), a Ag/AgCl/3 M KCl reference electrode, and a Pt mesh as counter electrode. Cyclic voltammetry and photochronoamperometric measurements were performed using a PGU-BI 100 potentiostat (IPS Jaissle). For illumination of the sample a He-Xe lamp (LC8 type 03, Hamamatsu Photonics) was used, integrating a red foil filter (No. 164 LEE Filters, λ > 600 nm) and with an incident power of 51 mW cm^{-2}, unless stated otherwise. Electrochemical impedance spectroscopy measurements were performed using an Autolab PGSTAT302N potentiostat (Metrohm-Autolab) in a 0.1 M KCl solution containing equimolar concentrations of $[Fe(CN)_6]^{4-}$ and $[Fe(CN)_6]^{3-}$ (5 mM each). The experiments were conducted at the equilibrium potential of the redox couple (DC potential of 236 mV vs. Ag/AgCl/3 M KCl) superimposed with an AC perturbation of 10 mV_{rms} amplitude in a frequency range comprised between 100 kHz and 100 mHz.

Gas Chromatography Detection

H_2 was detected using a gas chromatograph (GC, multiple gas analyzer #1, MG#1, SRI Instruments) equipped with a 3 meters HayeSep D column at 90 °C and a thermal conductivity detector (TCD). The detection with the modified sample was performed using a small-volume electrochemical cell (total volume: 180 µL, headspace: 50 µL), using N_2 to generate an inert atmosphere. After 36 minutes of a constant applied potential of 160 mV vs. SHE, an aliquot of 25 µL of the headspace was collected and manually on-column injected into the GC. For correlation of the retention time, a H_2 standard with a concentration of 800 ppm was injected by quadruplicate.

PSI monomer preparation

Thylakoid membrane preparation

20 L of a *T. elongatus* culture (grown in Airlift fermenter) were harvested and the thylakoid membranes were prepared as described previously.[7] The washed cells were incubated with buffer A (20 mM MES (pH 6.5), 10 mM $MgCl_2$, 10 mM $CaCl_2$), containing 0.2% (w/v) lysozyme, for 90 min at 37 °C and disrupted via Parr bomb (20 min, 4 °C, 2,000 psi). Cell debris and membrane systems were sedimented (10,000 rpm, 10 min, 4 °C), washed three times with buffer A, and subsequently with buffer B (20 mM MES (pH 6.5), 10 mM $MgCl_2$, 10 mM $CaCl_2$, 500 mM mannitol). Lastly, the cells were resuspended in buffer B with 20% (v/v) glycerol, frozen in liquid nitrogen, and stored at −80 °C.

PSI monomer isolation

Thylakoid membranes of *T. elongatus* were resuspended in buffer B and sedimented (30 min, 4 °C, 20,000 g, SS-34). Excess phycobilisomes were removed by subsequent washing with buffer B supplemented with 0.05% (w/v) DDM. The photosystem-containing pellet was resuspended in extraction buffer (20 mM HEPES pH 7.5, 10 mM $MgCl_2$, 10 mM $CaCl_2$, 200 mM ammonium sulfate (AMS), final chlorophyll concentration: 1 mg_{Chl} mL^{-1}), supplemented with 0.5% (w/v) LDAO and incubated at 20 °C for 30 min with gentle agitation. Samples were then sedimented via ultracentrifugation (60 min, 4 °C, 180,000 g) and the supernatant containing PSI was used for separation via discontinuous sucrose gradient (45 mL of 14% (w/v) sucrose in buffer B + 0.03% (w/v) DDM, underlaid with 5 mL 80% (w/v) sucrose cushion in buffer B + 0.03% (w/v) DDM, in a Ti-45 centrifugation tube; 18 h, 4 °C, 120,000 g, with slow acceleration and deceleration). After fractionation, green sections of the sucrose gradient were collected and mixed with 2 M AMS before application on a hydrophobic interaction chromatography column (POROS 50 OH, CV 53 mL, Thermo Fisher Scientific, USA) integrated in a high performance liquid chromatography (HPLC) system (ÄKTA purifier, GE Healthcare, Sweden). PSI monomers were eluted by a decreasing AMS gradient (1.65 M to 0 M AMS over 6 CV). Collected fractions were concentrated to a volume of 10 mL via an AMICON 8050 stirred cell (MWCO: 100 kDa, Ultracel-100 membrane, EMD Millipore Co., USA) and further applied on a desalting column in order to remove excess AMS (HiPrep 26/10 Desalting, GE Healthcare, Sweden).

Detergent exchange

Since LDAO in the monomer sample was suspected to interfere with the formation of PSI monolayers, a detergent exchange to DDM was carried out via ion exchange chromatography (IEC). 0.5 mg of isolated monomeric PSI were mixed with 30-fold excess of exchange buffer (20 mM HEPES pH 7.5, 10 mM $MgCl_2$. 10 mM $CaCl_2$, 500 mM mannitol, 1% (w/v) DDM) and incubated for 10 min before they were loaded on an IEC column (Uno Q6R, Bio Rad, Germany) integrated in the AKTA purifier system, previously described. The column was previously equilibrated (20 mM HEPES pH 7.5, 10 mM $MgCl_2$, 10 mM $CaCl_2$, 500 mM mannitol, 0.03% (w/v) DDM) and PSI was eluted via 130 mM $MgSO_4$ over 4 column volumes. PSI-containing fractions were washed via centrifuge concentrators (MWCO 100 kDa, Millipore®, Merck, USA) with equilibration buffer, concentrated to 2 mg PSI x mL^{-1} and flash-frozen in liquid N_2.

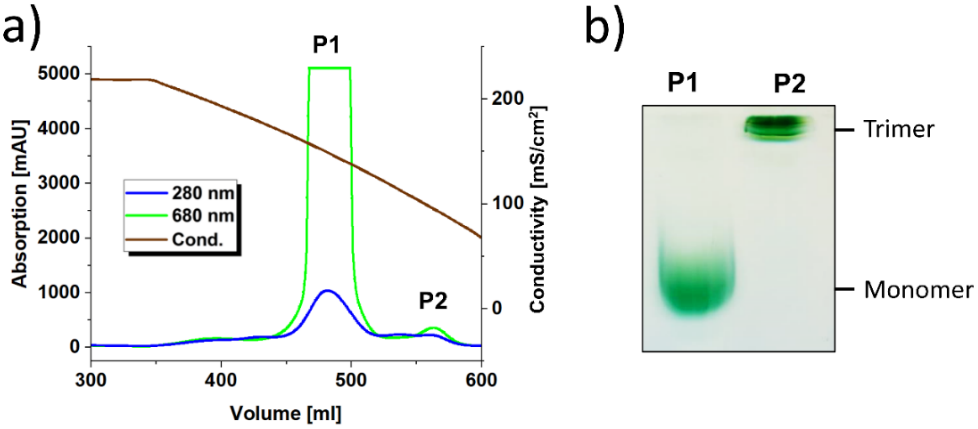

Figure S1. Isolation and characterization of monomeric PSI from *T. elongatus*. a) Thylakoid membrane extracts were loaded on a POROS 50 OH hydrophobic interaction column and separated with a linear ammonium sulphate gradient (2 M to 0 M). b) Samples of P1 and P2 (3.5 μg_{Chl} each) were analyzed by Blue Native (BN) PAGE to investigate the sample purity and species of PSI. P2 was further purified with an additional step of ion exchange chromatography before application on BN-PAGE.

Synthesis of BPEI-[CoCp2]

The synthesis of the cobaltocene-functionalized branched polyethyleneimine BPEI-[$CoCp_2$] (Scheme S1) was conducted as follows: 20 mg of branched polyethyleneimine (BPEI, 50 wt% in H_2O) were dried under reduced pressure at room temperature overnight. Subsequently, the dried BPEI was dissolved in 1 mL of dry DMSO under Ar-atmosphere and 12 µL of Ar-saturated triethylamine (Et_3N) were added and the solution was stirred for 5 min. After this, 12 mg (0.025 mmol) of 1-(2,5-dioxopyrrolidinylcarboxy)-cobaltoceniumhexafluorophosphate ([$CoCp_2$]-NHS, MCAT GmbH) were dissolved in 0.5 mL of dry DMSO and slowly added to the BPEI solution resulting in the solution to turn yellow. Subsequently, the solution was stirred for 5 h at room temperature. After the reaction was finished, 10 mL of diethyl ether were added to the solution, resulting in precipitation of a yellow oil. The product was washed with 2 × 10 mL of diethyl ether, 10 mL of dichloromethane, and 10 mL of methanol. The product was then dried at room temperature under reduced pressure. The dried product was dissolved in 2 mL of water. Subsequently, the polymer solution was subjected to dialysis by centrifugation against 0.1 M KCl over 5 kDa molecular weight cut-off membrane filters (8 × 20 min at 8,000 rpm) to replace the hydrophobic PF_6^- counterions with hydrophilic Cl^- counterions. Finally, the polymer was washed with H_2O by centrifugation over 5 kDa membrane filters (3 × 20 min at 8,000 rpm) to remove excess KCl, dissolved in 2 mL of H_2O obtaining a concentration of 13.5 mg mL^{-1}, and stored at 4 °C.

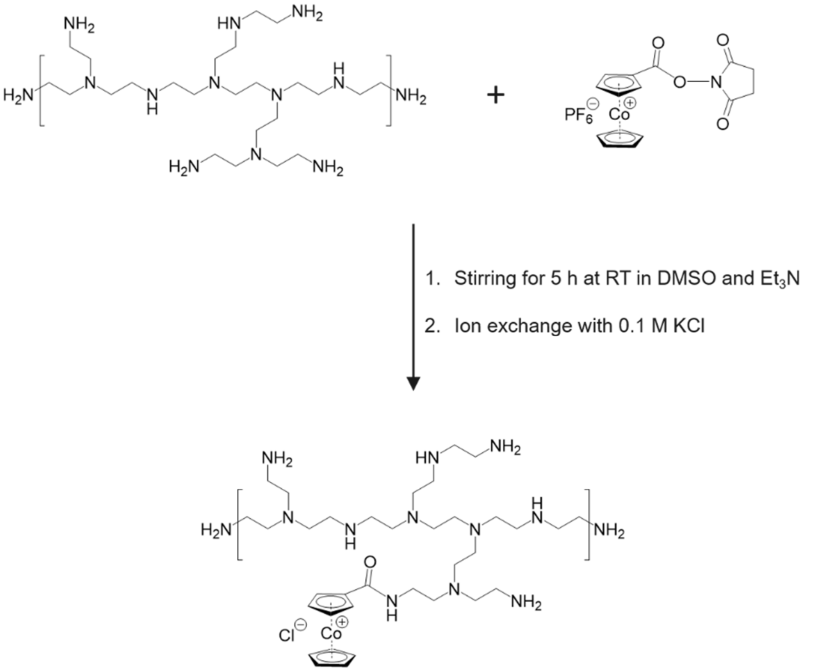

Scheme S1. Synthesis of the redox polymer BPEI-[$CoCp_2$]. After modification of branched polyethyleneimine with cobaltocene, the hydrophobic PF_6^- counterions were exchanged against the more hydrophilic Cl^- anions.

Additional Figures

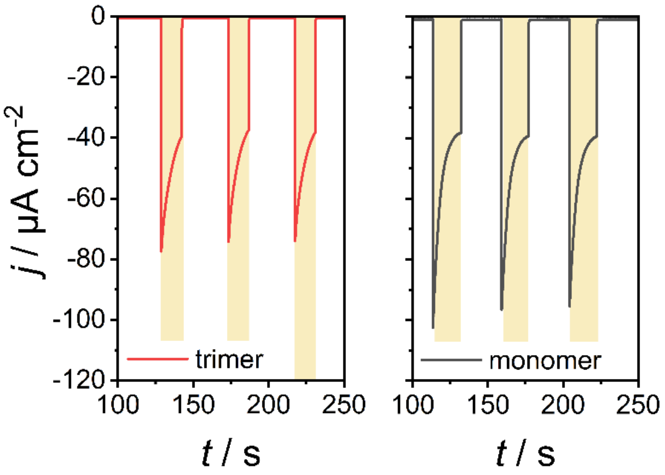

Figure S2. Photocurrent response for PSI/P-Os 3D films on Au electrodes. A mixture of isolated PSI trimers or monomers (chlorophyll concentration 0.27 mg mL^{-1}), P-Os (2.5 mg mL^{-1}), and PEGDGE as cross-linker (0.04 mg mL^{-1}) was drop cast over each electrode (Ø = 2 mm). E_{app} = 210 mV vs. SHE. Electrolyte: 2 mM MV^{2+} in air-equilibrated 150 mM phosphate-citrate buffer, pH 4.0. Illumination with red light (36 mW cm^{-2}) during the times indicated by the yellow boxes.

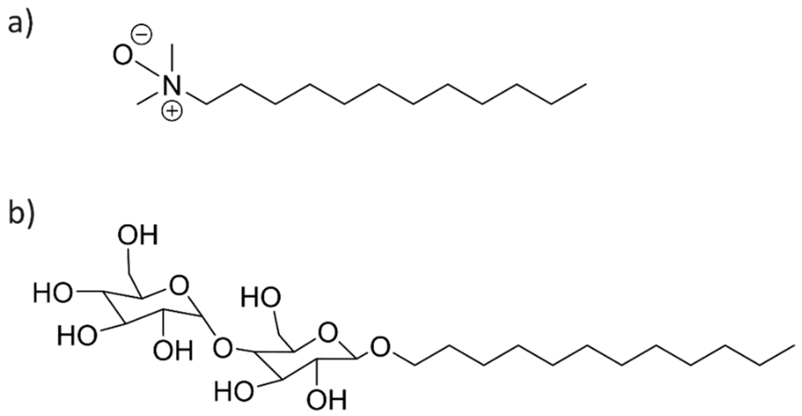

Figure S3. Comparison of the chemical structures of a) lauryldimethylamine oxide (LDAO) an ionic amphoteric surfactant and b) *n*-dodecyl β-D-maltoside (DDM) a non-ionic surfactant.

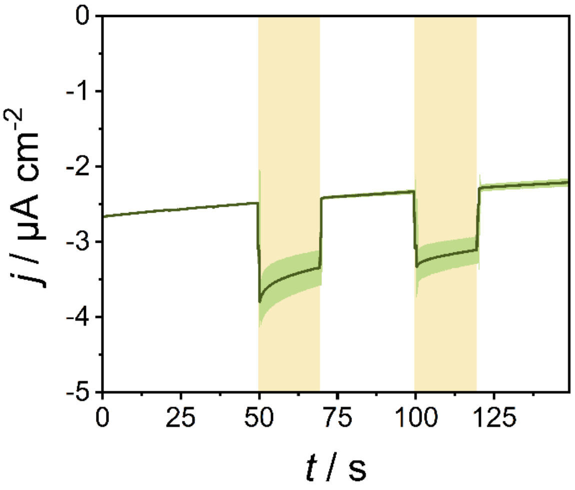

Figure S4. Average photocurrent response for electrodes fabricated with a monolayer of PSI monomers deposited over Au substrates. The green shaded region indicates the standard deviation of the measurements (N = 3). E_{app} = 210 mV vs. SHE. Electrolyte: 2 mM MV^{2+} in air-equilibrated 150 mM phosphate-citrate buffer, pH 4.0. Illumination with red light (51 mW cm^{-2}) during the times indicated by the yellow boxes.

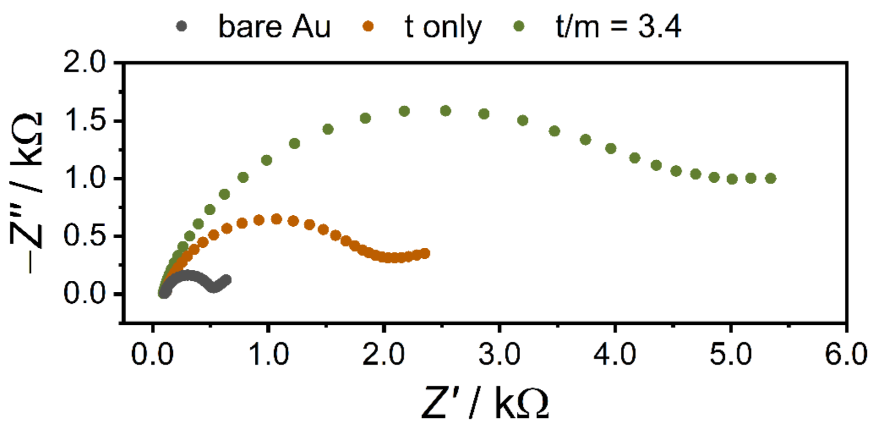

Figure S5. EIS characterization of PSI-LB monolayers consisting of only PSI trimers (t only) or a mixture of trimers and monomers in optimal ratio (t/m) deposited over Au substrates. $[Fe(CN)_6]^{4-}/[Fe(CN)_6]^{3-}$ (5 mM each) in 0.1 M KCl, dark conditions. The same batch of Au wafer was used for all measurements.

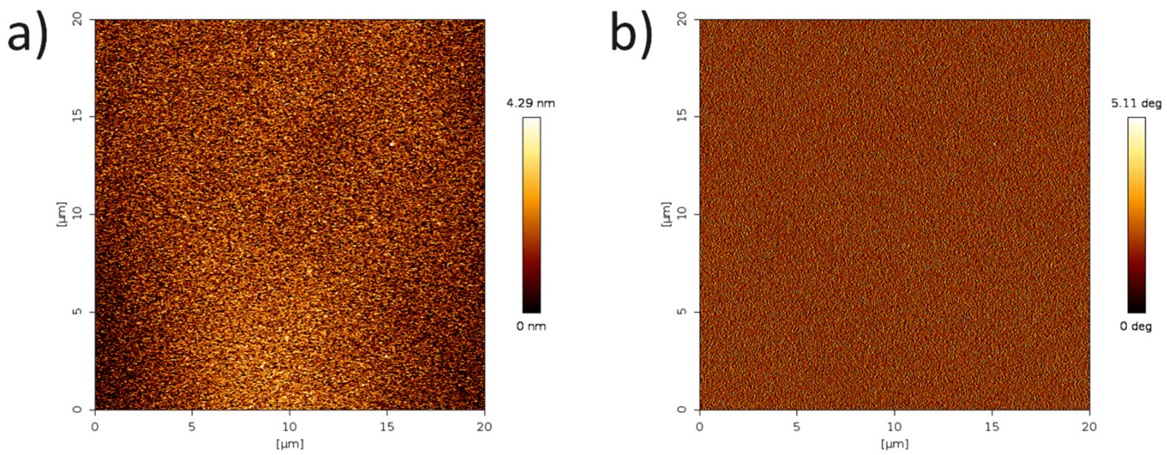

Figure S6. AFM characterization of P-Os modified electrodes: a) height profile, b) phase image. Measurements performed in the AC mode at a frequency of 75 kHz, using a monolithic silicon AFM probe with a constant force of 3 N m^{-1}.

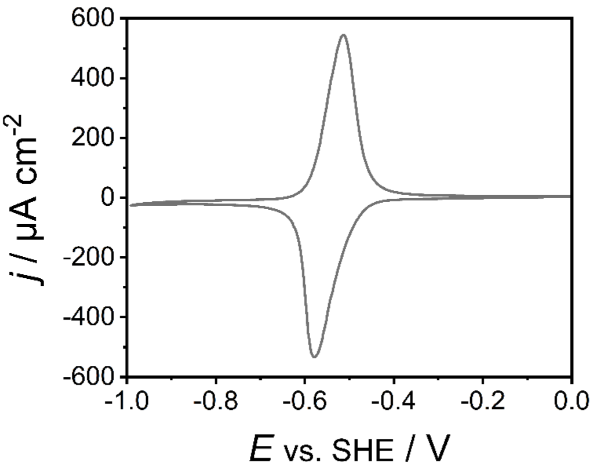

Figure S7. Electrochemical characterization of the cobaltocene modified polymer (BPEI-[$CoCp_2$]), revealing a midpoint potential of −547 mV vs. SHE. Cyclic voltammogram for 5 µL of BPEI-[$CoCp_2$] (13.5 mg mL^{-1}, in H_2O) drop cast over a glassy carbon electrode (3 mm diameter). Scan rate: 10 mV s^{-1}. Ar-saturated 0.1 M phosphate buffer pH 7.4.

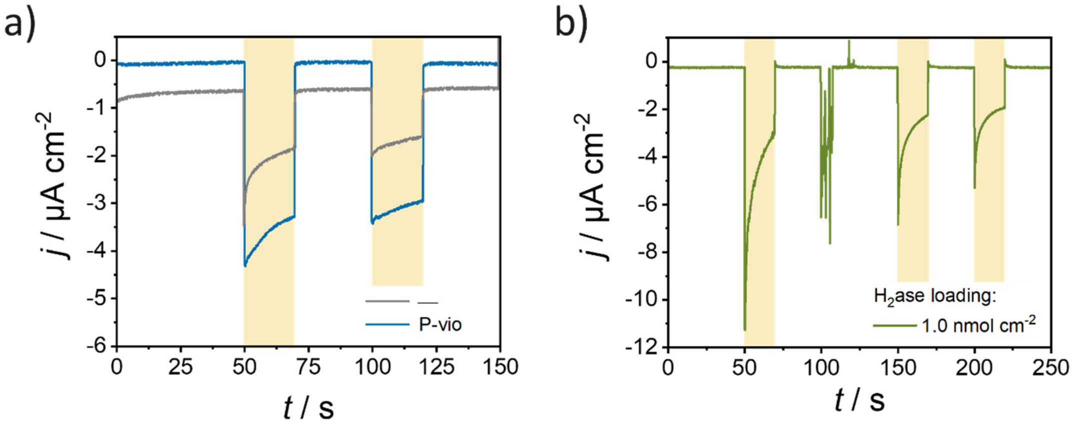

Figure S8. a) Photocurrent response obtained for PSI-LB/P-Os/Au in the absence or presence of a top modification layer consisting of P-vio (loading: 122 µg cm^{-2}). Electrolyte: air-equilibrated 150 mM phosphate-citrate buffer, pH 5.6. E_{app} = 210 mV vs. SHE. Illumination with red light (51 mW cm^{-2}) during the times indicated by the yellow boxes. b) Photochronoamperometric response for PSI-LB/P-Os/Au incorporating a top layer consisting of H_2ase/P-vio, with a H_2ase nominal loading of 1.0 nmol cm^{-2} and 122 µg cm^{-2} of P-vio. E_{app} = 210 mV vs. SHE. Illumination with white light at an incident power of 113 mW cm^{-2} during the times indicated by the yellow boxes. Ar-saturated 150 mM phosphate-citrate buffer pH 5.5. *The signal at $t \approx 100$ s is noise associated to an increased bubbling of Ar.

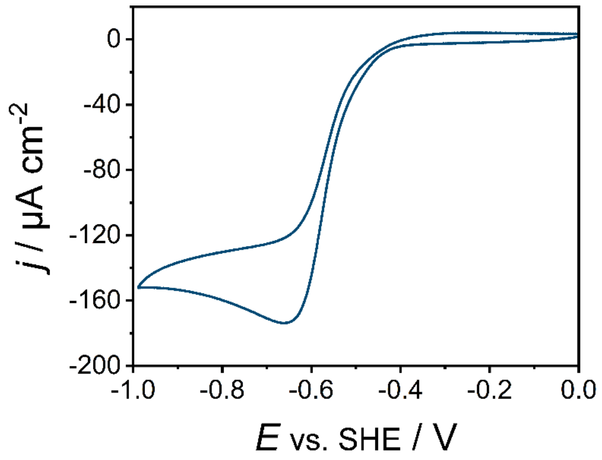

Figure S9. Investigation of H_2 evolution catalyzed by [NiFeSe]-H_2ase embedded in the BPEI-[$CoCp_2$] redox polymer. Cyclic voltammogram for a mixture of 1 µL BPEI-[$CoCp_2$] (13.5 mg mL^{-1}, in H_2O) and 1.5 µL of [NiFeSe]-H_2ase (135 µM) drop cast over a glassy carbon electrode (3 mm diameter). Scan rate: 10 mV s^{-1}. Ar-saturated 0.1 M phosphate buffer pH 7.4.

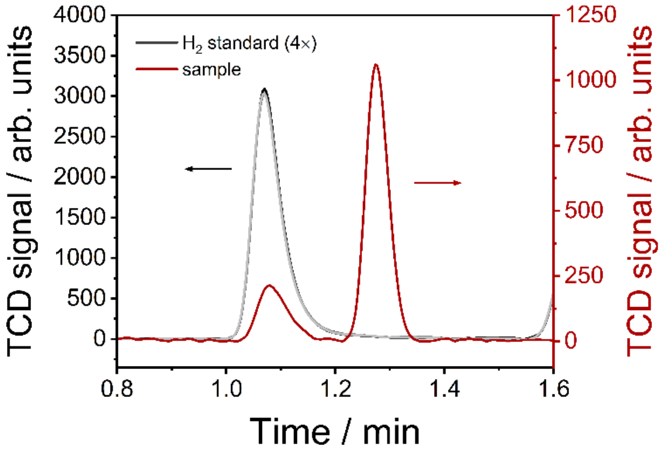

Figure S10. Gas chromatography analysis for a PSI-LB/P-Os/Au electrode with a top H_2ase/BPEI-[$CoCp_2$] layer (nominal loading of H_2ase: 0.16 nmol cm^{-2}, redox polymer loading: 107 µg cm^{-2}). Electrolyte: N_2-saturated 150 mM phosphate-citrate buffer, pH 5.6. E_{app} = 160 mV vs. SHE. An aliquot of 25 µL of the headspace was injected into the GC after 2200 s of illumination using white light at an incident power of 113 mW cm^{-2}. A H_2 standard (800 ppm) injected by quadruplicate was used for correlation of the retention time. The signal at about 1.3 min for the sample corresponds to O_2 due to possible traces in the electrochemical cell and the use of a manual injection.

References

[1] T. Kothe, S. Pöller, F. Zhao, P. Fortgang, M. Rögner, W. Schuhmann, N. Plumeré, *Chem. Eur. J.* **2014**, *20*, 11029.

[2] F. Zhao, P. Wang, A. Ruff, V. Hartmann, S. Zacarias, I. A. C. Pereira, M. M. Nowaczyk, M. Rögner, F. Conzuelo, W. Schuhmann, *Energy Environ. Sci.* **2019**, *12*, 3133.

[3] a) F. Conzuelo, N. Marković, A. Ruff, W. Schuhmann, *Angew. Chem. Int. Ed.* **2018**, *57*, 13681; b) F. Conzuelo, N. Marković, A. Ruff, W. Schuhmann, *Angew. Chem.* **2018**, *130*, 13870.

[4] C. Tapia, R. D. Milton, G. Pankratova, S. D. Minteer, H.-E. Åkerlund, D. Leech, A. L. De Lacey, M. Pita, L. Gorton, *ChemElectroChem* **2017**, *4*, 90.

[5] A. Badura, D. Guschin, T. Kothe, M. J. Kopczak, W. Schuhmann, M. Rögner, *Energy Environ. Sci.* **2011**, *4*, 2435.

[6] M. C. Marques, C. Tapia, O. Gutiérrez-Sanz, A. R. Ramos, K. L. Keller, J. D. Wall, A. L. de Lacey, P. M. Matias, I. A. C. Pereira, *Nat. Chem. Biol.* **2017**, *13*, 544.

[7] H. Kuhl, J. Kruip, A. Seidler, A. Krieger-Liszkay, M. Bünker, D. Bald, A. J. Scheidig, M. Rögner, *J. Biol. Chem.* **2000**, *275*, 20652.
